# Phase diagram of the layered oxide SnO: GW and electron-phonon studies

**DOI:** 10.1038/srep16359

**Published:** 2015-11-10

**Authors:** Peng-Jen Chen, Horng-Tay Jeng

**Affiliations:** 1 Department of Physics, National Taiwan University, Taipei 10617, Taiwan; 2 Nano Science and Technology Program, Taiwan International Graduate Program, Academia Sinica, Taipei 11529, Taiwan and National Taiwan University, Taipei 10617, Taiwan; 3 Institute of Physics, Academia Sinica, Taipei 11529, Taiwan; 4 Department of Physics, National Tsing Hua University, Hsinchu 30013, Taiwan

## Abstract

First-principles calculations are performed to study the electronic properties and the electron-phonon interactions of the layered oxide semiconductor SnO. In addition to the high hole mobility that makes SnO a promising material in electronics, it has recently been reported that the semimetallic phase under pressure is superconducting. The superconducting *T*_*c*_ curve exhibits a dome-like feature under pressure and reaches the maximum of 1.4 K at *p* = 9.2 GPa. Both its crystal structure and the dome-like *T*_*c*_ curve are reminiscent of the Fe-based superconductor FeSe. Motivated by this observation, we investigate the electronic, phonon, and their interactions in SnO using first-principles schemes. GW approximation is adopted to correct the underestimated band gaps, including real and continuous band gaps in the semiconducting and semimetallic phases. The phase diagram showing the semiconductor-to-semimetal transition and the *T*_*c*_ curve has been successfully reproduced. Detailed analysis of the electron-phonon interactions demonstrate the importance of the out-of-plane motions of O atoms and the Sn-*s* lone pairs for the superconductivity to occur. Our method combining GW and e-ph calculations can be further extended to the study of other materials that undergo insulator-to-superconductor phase transition.

Oxide semiconductors play an important role in device applications. Due to the large effective mass of holes (fully occupied O-*p* bands), most of the known oxide semiconductors are n-type. To promote the performance of the oxide-based devices, p-type semiconductors are of equal importance. Among the few known p-type oxide semiconductors, SnO has attracted much attention due to its high hole mobility^1^,^2^. Besides, conversion into the n-type semiconductor can be achieved upon doping with Y or Sb, making SnO a bipolar conducting oxide^2^. SnO has great potential in applications such as catalysis^3^, coating^4^, and precursor for the production of SnO_2_^5^. It is also suggested that SnO exhibits high capacity as a lithium-ion storage material^6^. In addition to the fascinating electronic applications, superconductivity in SnO has also been observed under pressure^7^. Interestingly, the crystalline structure of SnO is the same as the intensively studied Fe-based superconductor (FeSC) FeSe^8^. Furthermore, SnO and FeSe exhibit similar dome-like *T*_*c*_ curve under pressure and show nesting between the electron- (*M*) and hole- (Γ) pockets. Taken together, the superconductivity in SnO is appealing for its possible connection to that in FeSe.

Both SnO and FeSe have *α*-PbO layered structure as shown in Fig. 1 (space group *P*4/*nmm*, no. 129). FeSe is a superconductor with *T*_*c*_ = 8 K^8^. Upon the application of pressure, its *T*_*c*_ increases up to 37 K at *p* ~ 7 GPa^9^,^10^. Although there are experimental and theoretical studies showing the unconventional superconductivity in the FeSCs^11^,^12^,^13^,^14^,^15^, the Fe isotope effect has been observed^16^,^17^,^18^,^19^,^20^ which implies the role of phonons may not be sorely excluded. On the other hand, SnO is comprised of nonmagnetic atoms so magnetism-related mechanism can not account for the electron pairing in SnO. It is thus natural to speculate that the superconductivity is caused by the electron-phonon (e-ph) couplings.

The superconductivity in SnO occurs under pressure. Under ambient condition, SnO is a semiconductor with an indirect gap (Γ-**M**) of 0.7 eV^21^ and a direct gap of 2.5 ~ 3.0 eV^22^. The band gap closes when exposed to an external pressure of about 5 GPa at room temperature^23^,^24^. It becomes superconducting under the pressure between 5.8 and 16.2 GPa, with the maximum *T*_*c*_ ~ 1.4 K at *p* = 9.2 GPa^7^. Structural phase transition into the orthorhombic or monoclinic phase is observed at *p* = 16 ~ 17 GPa^24^. In this work we aim at the elucidation of the e-ph interactions in SnO and therefore focus on the pressures in the range between 5.8 and 16.2 GPa within which SnO shows superconductivity.

The first problem encountered when studying the electronic properties of SnO is the well-known band gap issue in density functional theory (DFT). In the case of SnO that undergoes semiconductor-to-semimetal transition, the “band gap” is generalized to the relative energy between the electron- and hole- pockets. Thus, a “negative band gap”, also referred to as the band overlap, denotes the semimetallic state. Incorrect “band gap” may cause problems not only in the macroscopic state (semiconducting or semimetallic) but also in the sizes of the electron- and hole- pockets in the semimetallic phase. The latter will further lead to errors in the e-ph coupling calculations. To overcome this problem, we resort to the GW approximation (GWA)^25^,^26^ to correct the band energies. With the quasiparticle effects taken into account, GWA has proven its success in reproducing the band gaps of a wide range of materials^27^,^28^,^29^. Having obtained the correct band overlaps, we proceed to the e-ph calculations for the *T*_*c*_ curve with respect to the pressure. The full *T*_*c*_ curve of SnO is reproduced from our e-ph calculations, which demonstrates the fact that the superconductivity in SnO can be explained within the framework of the BCS theory.

## Results

**Figure 2:**
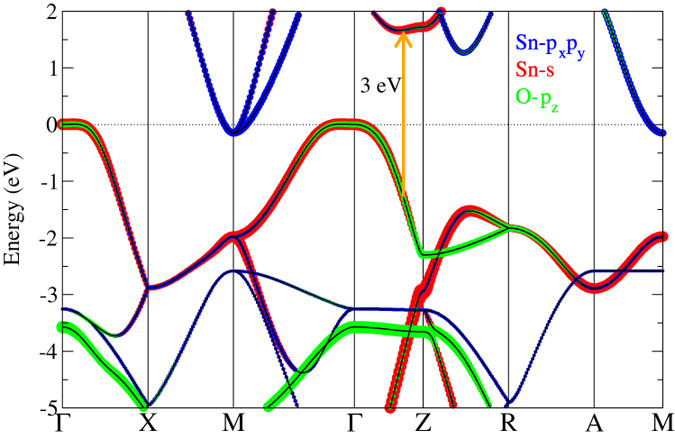
Band structure of SnO that yields maximum *T*_*c*_ = 1.5 K. Blue, red, and green circles represent the components of Sn-*p*_*x*_*p*_*y*_, Sn-*s*, and O-*p*_*z*_ orbitals, respectively. The band overlap between Γ and M is about 0.15 eV. The arrow marks the primary optical transitions that giving rise to the peak in 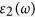 in Fig. 5.

Our DFT calculations show that SnO has a small indirect gap (<0.1 eV) under ambient condition and becomes semimetallic at *p* ~ 1 GPa. These results are in agreement with the previous DFT calculations^23^,^30^, but disagree with the experiments^21^,^22^. The underestimation of the band gap is a well-known issue of DFT originating from the simplified one-electron picture. To obtain more accurate results, we apply the GWA to correct the band energies. At zero pressure, the direct and indirect band gaps increase to 2.88 and 0.72 eV, respectively, from the GWA calculations. These results are consistent with a previous work that studies the band gap of SnO under ambient condition with GWA^31^. In our work, we further investigate the effect of pressure on the band gap of SnO. The GWA corrected indirect gap closes at *p* ~ 5.3 GPa. The excellent agreement with the experiment transition pressure around 5 GPa^23^,^24^ reveals the success of the GWA in reproducing the pressure effect on the band gaps in SnO. The results with the extension into the semimetallic phase are shown in Table 1. When becoming semimetallic, the topology of the Fermi surface exhibits a hole-pocket around Γ and an electron-pocket around **M**. The band structure of the semimetallic SnO is shown in Fig. 2. As reported previously in other works^7^,^23^,^24^, the hole-pocket is mostly of Sn-*s* and O-*p*_*z*_ characters, whereas the electron-pocket consists mainly of the Sn-*p*_*x*_*p*_*y*_ components. We also note that these two pockets show rigid shift in energy as the pressure varies, namely, the dispersion and components of the bands remain unchanged. The Sn-*s* electrons form chemically inactive lone pairs accumulating in the regions away from the Sn-O-Sn slabs. The lone pairs are considered critical in Sn monochalcogenides for their effects on the structural stability^23^,^32^. As the applied pressure increases to 16 GPa, reduced interlayer spacing causes hybridization between the lone pairs from two adjacent layers and structural instability then occurs^24^.

**Figure 3:**
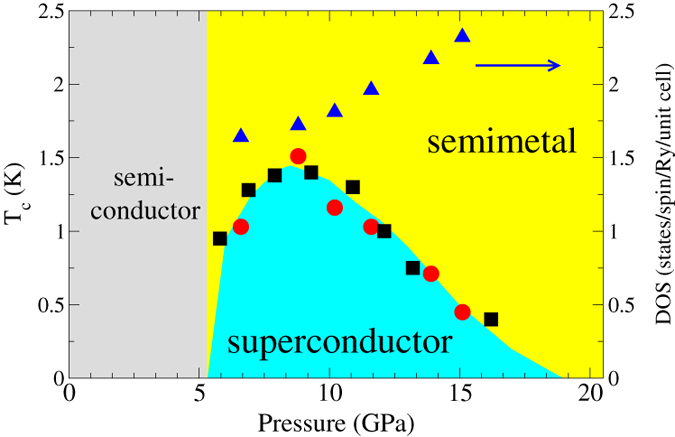
Phase diagram of SnO. The painted areas in colors that denote different phases are derived from experiment and the black squares display the experimental data of *T*_*c*_^7^. The values of *T*_*c*_ from our calculations are represented by the red circles. The blue triangles represent the density of states at the Fermi level *N*_*F*_.

The main task of this work is to study the variation of *T*_*c*_ with respect to the pressure. Our e-ph calculations show that the *T*_*c*_ reaches its maximum of 1.5 K (λ = 0.52) when the band overlap is 0.15 eV, which corresponds to rescaled pressure *p* ~ 8.8 GPa by interpolating the GWA band overlaps (see Table 1). The phase diagram is shown in Fig. 3. The calculated variation of *T*_*c*_ with respect to the pressure agrees well with the experimental observations^7^ that are represented in black squares. It is thus apparent that SnO is a BCS-type superconductor, although it assumes some common features with the Fe-based superconductor FeSe. Both experimentally and theoretically, the *T*_*c*_ drops to zero at *p* > 16.2 GPa^7^, which might be attributed to the structural phase transition that takes place at high pressure. Structural phase transition from tetragonal to orthorhombic or monoclinic structures is observed at *p* ~ 17.5 GPa^24^.

**Figure 4:**
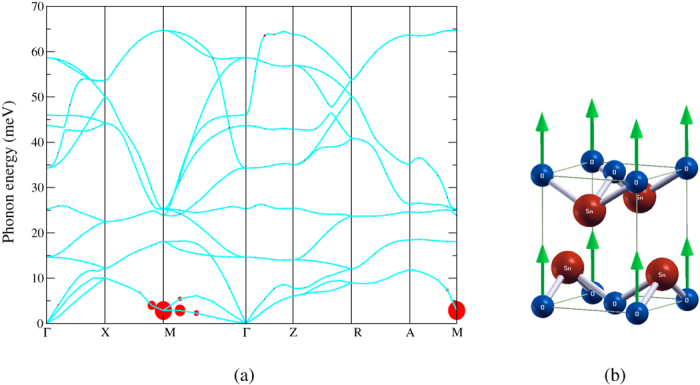
(**a**) Phonon spectrum of SnO when *T*_*c*_ reaches maximum (band gap overlap ~0.15 eV). The size of the red dots represents the magnitude of the electron-phonon coupling 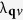. (**b**) Atomic displacement pattern of the lowest-energy phonon mode at **q** = **M**. This mode mainly involves the vertical motions of the O atoms. The Sn atoms shift horizontally from their equilibrium positions, away from the approaching O atoms. The displacements, however, are too small to be visible in this figure. Note also that the phase difference of the displacements due to the zone-boundary phonon is not displayed in the figure.

The phonon spectrum as well as the strength of the e-ph coupling 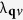 when *T*_*c*_ = 1.5 K is shown in Fig. 4(a). We can clearly see that the e-ph couplings almost come from the pairing of the electrons mediated by the low-energy phonons with momenta **q** around **M**. In other words, 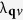 is highly non-uniform throughout the BZ and therefore a dense **q**-grid is needed to obtain the converged value of *T*_*c*_. Failure to appropriately catch the information around M will result in errors in the total e-ph coupling λ, and hence *T*_*c*_. A multigrid described in the **Methods** section is used to achieve the balance between accuracy and the available computational resources. The displacement patterns of the lowest-energy phonon mode with **q** = **M** involve the out-of-plane motions of O atoms as shown in Fig. 4(b). Because these two modes are degenerate, only one of them is shown in the figure. The other mode corresponds to the vibration of the other O atom moving in the same way.

## Discussion

The e-ph coupling 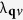 is computed by using equations (1)–(3), 8, 10 shown in the following.


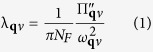


where *N*_*F*_ is the density of states (DOS) at the Fermi level, 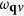 is the phonon frequency of mode 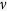 at wave vector **q**. The e-ph quasiparticle linewidth, 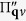, is given by


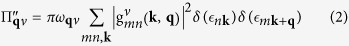



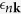
 is the energy of the KS orbital and the dynamical matrix reads


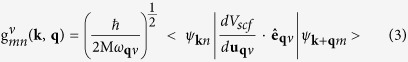


where 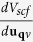 represents the variation of the KS potential with respect to the small atomic displacement 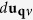 of the given phonon mode. M and 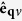 denote the mass of the atom and the unit vector along 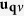, respectively. The *T*_*c*_ can then be estimated by the McMillan formula:


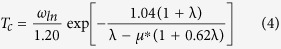


where


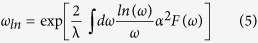



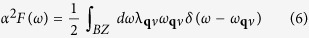


**Figure 5:**
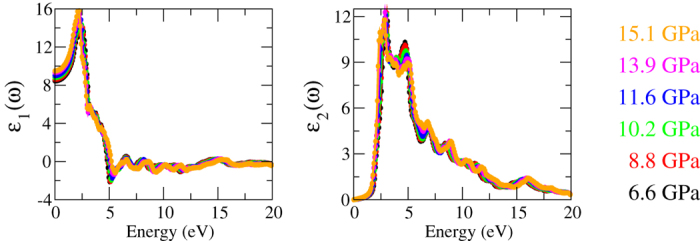
The dielectric function *ε*(*ω*) at pressures ranging from 6.6 to 15.1 GPa. The insignificant distinction between the *ε*(*ω*) at different pressures indicates that the screening effect can approximately be regarded as unchanged as pressure varies.

The total e-ph coupling constant 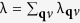. It can be seen that the ground state band energies and wave functions are used as the input to calculate the phonon-related properties. This means appropriate ground state properties are required to obtain the accurate phonon-related quantities. However, the sizes of the Fermi surfaces calculated from DFT are problematic owing to the incorrect description of the relative energies between the electron- and hole- pockets as mentioned previously. Although the GWA amends the problem, the inclusion of GWA in the e-ph calculations is not practical. Therefore, a multi-step way is adopted to study the evolution of *T*_*c*_ with respect to the pressure. The rigid shift in energy of the pockets under the application of pressure manifests that the band overlap can be regarded as a parameter to represent the effect of pressure. For this reason, we turn to study *T*_*c*_ versus the band overlap, 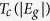. That is, the *T*_*c*_ is calculated at the DFT level using the structural parameters observed in the experiment^24^, while the pressure is rescaled by the GWA corrected band overlaps as explained below. Owing to the improper correspondence between the DFT band overlap and the pressure, we interpolate the GW corrected band overlaps in Table 1 to obtain the “true” pressure that has this DFT band overlap in magnitude. By virtue of this process, 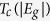 can be converted to 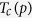 and then the phase diagram is obtained with the rescaled pressure as shown in Fig. 3. The validity of this procedure is based on two reasons stated below. First, it is generally believed that the delocalized *s*- and *p*− orbitals are well described at the DFT level. GWA provides insignificant corrections to the orbitals^26^,^33^,^34^,^35^. What GWA does for the *sp*-system is to calculate the quasiparticle energies, from which the band gap can be extracted. Second, in SnO the pressure dependence of the screening effect is weak. Figure 5 shows the dielectric function 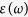 at different pressures. The similarity of 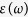 indicates that the effect of the applied pressure can be restricted to the energy shift of the two pockets.

**Figure 6:**
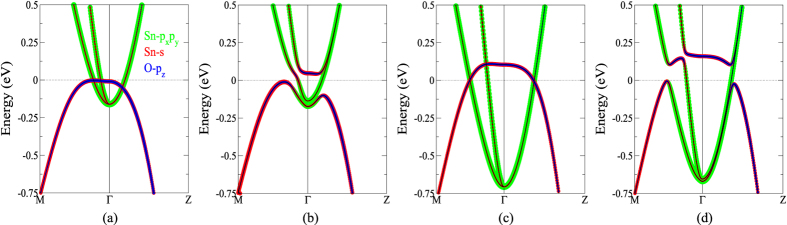
Band structures of SnO in a 2 × 2 × 1 supercell with frozen phonons (see Fig. 4(b) **for the atomic displacements).** (**a**,**b**) represent the band structures when *T*_*c*_ = 1.5 K with undistorted and distorted crystal structures, respectively. Shown in (**c**,**d**) are the band structures corresponding to *T*_*c*_ = 0.5 K. Color representations are shown in (**a**).

A detailed analysis based on frozen phonons has also been carried out to get an insightful view on the origin of superconductivity in SnO. As shown in Fig. 3, apparently the dome-like *T*_*c*_ curve can not be explained solely by the increasing *N*_*F*_ resulting from the growing sizes of both the electron- and hole- pockets with the raising pressure. We therefore attribute the decrease of *T*_*c*_ at high pressures to the diminished dynamical matrix elements. In Fig. 4(a) we see that the e-ph coupling is largest at **q** = **M**. With a nonzero phonon momentum, there is a phase difference of the atomic displacements between two different cells. Therefore, a supercell is required to accommodate all atoms vibrating with different phases to retain the periodicity. When **q** = **M**, the atoms in two adjacent cells along both x and y directions are moved in opposite directions with equal displacement for the phase difference is *π*. Although a 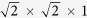 supercell suffices in the frozen phonon study to take care of the phase of the atomic vibrations at **q** = **M**, the slight horizontal displacements of Sn make the unrotated cell a better choice. Therefore, a 2 × 2 × 1 supercell is used in our frozen phonon calculations for simplicity. The zoomed-in band structures of the undistorted and distorted lattice structures are shown in Fig. 6. The corresponding *T*_*c*_’s are 1.5 K and 0.4 K in Fig. 6(a–d), respectively. In a 2 × 2 × 1 supercell, the electron-pocket folds back to Γ. A prominent difference is seen around Γ near the Fermi level. Crossing of bands without changing the orbital characters around the crossing points can be clearly seen in Fig. 6(a,c) for the undistorted crystals, indicating very week interactions between the hole and electron pockets. When distorted by the M-point phonon as shown in Fig. 6(b,d), orbital re-hybridizations take place near the crossing points. The electron-pockets originally of Sn-*p*_*x*_*p*_*y*_ characters markedly mix with the Sn-*s* and O-*p*_*z*_ orbitals of the hole-pocket. At the optimal pressure (Fig. 6(b)), the electron- and hole- pockets interact with each other right around the Fermi level. Due to the lifting of energy above the Fermi level, the originally occupied states around Γ (the flat region) become unoccupied. The remarkable change of the states in energy, which alters the occupation and orbital characters and thus yields spatial redistribution of charge, indicates a large 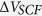. The strong orbital re-hybridizations, charge fluctuations, and the large deformation potentials 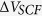, altogether maximize the *T*_*c*_ (1.5 K). As the pressure increases (Fig. 6(d)), the interacting points (orbital re-hybridization region) move away from the Fermi level and weaken the effect on *T*_*c*_. Since the re-hybridized bands driven by the M-phonon are mostly unoccupied, the resulting charge fluctuations become small and thus the deformation potential 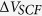 is smaller. This explains why the dynamical matrix elements diminish and the contributions to *T*_*c*_ (0.4 K) are reduced correspondingly at high pressure.

**Figure 7:**
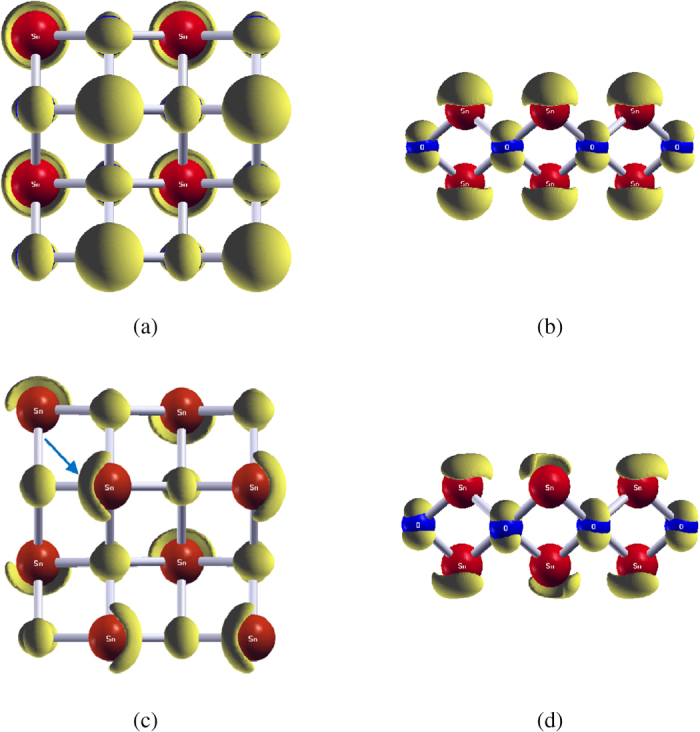
Charge density at the Fermi level in SnO when *T*_*c*_ = 1.5 K. A 2 × 2 × 1 supercell is used in the frozen phonon calculations. (**a**,**b**) show the top (001) and side (110) views of the charge density with undistorted crystal, while (**c**,**d**) display those with the distorted crystal. One of the Sn-*s* lone pairs that fluctuate remarkably is marked by an arrow in (**c**), while the charges around O atoms (blue spheres) show insignificant change.

Figure 7 displays the charge density at the Fermi level of the structure that gives *T*_*c*_ = 1.5 K. Panels (a,b) and (c,d) correspond to the undistorted and distorted crystals, respectively. The Sn-*s* and O-*p*_*z*_ natures of the charges can obviously be seen. The charge distributions around O atoms barely change when the crystal is distorted, while discernible charge fluctuations are seen in the Sn-*s* lone pairs. The upward (downward) motion of the O atoms expels the lone pair electrons of the upper (lower) Sn atoms. This implies the importance of the Sn-*s* lone pairs in the electron pairing in SnO. In short, our results indicate that while the key phonons involve the motions of the O atoms, the Sn-*s* lone pair electrons are the main participants in the electron pairs that give rise to the superconductivity.

## Conclusions

To conclude, with the aid of the GWA we reproduce the band gap in ambient condition as well as the onset pressure at which SnO becomes semimetallic. The curve of *T*_*c*_ versus pressure obtained from our e-ph calculations agrees well with the experiment. The *T*_*c*_ has the maximum of about 1.5 K at *p* ~ 8.8 GPa and goes down to ~0.5 K at *p* ~ 15 GPa. The frozen-phonon supercell calculations indicate that the charge fluctuation of the Sn-*s* lone pairs is crucial for the occurrence of the superconductivity in SnO. Our results reveal that SnO is a conventional BCS-type superconductor and that the e-ph interactions originate mainly from the coupling between the out-of-plane vibrations of the O atom and Sn-*s* lone pair electrons. Our works not only give a thorough investigation on SnO, but also provide technical treatment in studying the insulator-to-superconductor transition. Informative connection can also be made to elucidate the role of phonons in FeSe.

## Methods

The Kohn-Sham (KS) ground state and the phonon calculations are performed using the QUANTUM ESPRESSO code^36^ with the generalized gradient approximation (GGA) pseudopotentials. The cutoff energy of 60 Ry and 18 × 18 × 18 Monkhorst-Pack k-point mesh are used in the calculations with the lattice structures under pressures obtained from experiments^24^. The BerkeleyGW code^26^,^37^ is used to perform the GW calculations. The phonon frequencies are calculated within the framework of the many-body perturbation theory^38^ based on the KS ground states. XCrySDen is used for the crystal and charge density visualization^39^.

The BZ in **q**-space is sampled by a 3 × 3 × 3 grid, except for the regions around M and along Γ-M direction where a 9 × 9 × 6 grid is used. Ignorable contributions of 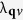 from outside the fine grid, as Fig. 4(a) depicts, make it reasonable to approximate the **q**-space summation by a coarse grid without loss of accuracy.

## Additional Information

**How to cite this article**: Chen, P.-J. and Jeng, H.-T. Phase diagram of the layered oxide SnO : GW and electron-phonon studies. *Sci. Rep.* **5**, 16359; doi: 10.1038/srep16359 (2015).

## Figures and Tables

**Figure 1:**
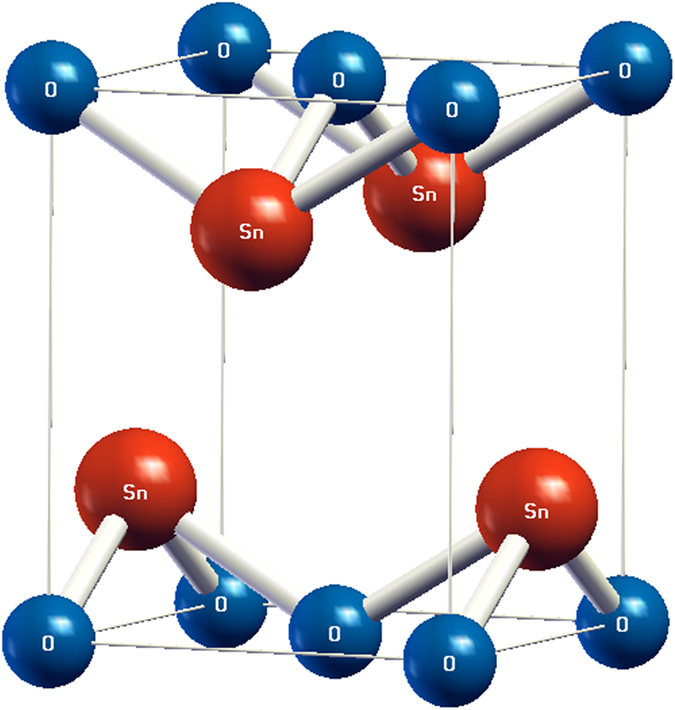
Crystal structure of SnO (*P*4/*nmm*). The lattice parameters are: a = 3.80 Å, c/a = 1.2744, and *z*_*Sn*_ = 0.2389 at ambient pressure^24^. The blue and red spheres represent the O and Sn atoms, respectively.

**Table 1:** Band gaps (in eV) at different pressures (GPa) obtained from GGA and GWA.

*p*	0.0	5.1	8.0	9.2	10.0	11.7	13.3	15.1
GGA	0.07	−0.56	−0.76	−0.93	−1.02	−1.18	−1.34	−1.28
GW	0.72	0.03	−0.07	−0.19	−0.22	−0.38	−0.54	−0.81

Negative values indicate the semimetallic phase. By extrapolation of the GW results the gap closes at *p* *~* 5.3 GPa, which agrees well with the experimental observations.
